# Technological quality requirements for stereotactic radiotherapy: Expert review group consensus from the DGMP Working Group for Physics and Technology in Stereotactic Radiotherapy

**DOI:** 10.1007/s00066-020-01583-2

**Published:** 2020-03-24

**Authors:** Daniela Schmitt, Oliver Blanck, Tobias Gauer, Michael K. Fix, Thomas B. Brunner, Jens Fleckenstein, Britta Loutfi-Krauss, Peter Manser, Rene Werner, Maria-Lisa Wilhelm, Wolfgang W. Baus, Christos Moustakis

**Affiliations:** 1 Klinik für Radioonkologie und Strahlentherapie, National Center for Radiation Research in Oncology (NCRO), Heidelberger Institut für Radioonkologie (HIRO), Universitätsklinikum Heidelberg, Heidelberg, Germany; 2 Klinik für Strahlentherapie, Universitätsklinikum Schleswig-Holstein, Kiel, Germany; 3 Klinik für Strahlentherapie und Radioonkologie, Universitätsklinikum Hamburg-Eppendorf, Hamburg, Germany; 4 Abteilung für Medizinische Strahlenphysik und Universitätsklinik für Radio-Onkologie, Inselspital—Universitätsspital Bern, Universität Bern, Bern, Switzerland; 5 Universitätsklinik für Strahlentherapie, Universitätsklinikum Magdeburg, Magdeburg, Germany; 6 Klinik für Strahlentherapie und Radioonkologie, Universitätsmedizin Mannheim, Universität Heidelberg, Mannheim, Germany; 7 Klinik für Strahlentherapie und Onkologie, Universitätsklinikum Frankfurt, Frankfurt am Main, Germany; 8 Institut für Computational Neuroscience, Universitätsklinikum Hamburg-Eppendorf, Hamburg, Germany; 9 Klinik für Strahlentherapie, Universitätsmedizin Rostock, Rostock, Germany; 10 Klinik für Radioonkologie, CyberKnife- und Strahlentherapie, Universitätsklinikum Köln, Cologne, Germany; 11 Klinik für Strahlentherapie—Radioonkologie, Universitätsklinikum Münster, Münster, Germany

**Keywords:** Radiosurgery, Recommendations, Technique, SRS, FSRT, SBRT

## Abstract

This review details and discusses the technological quality requirements to ensure the desired quality for stereotactic radiotherapy using photon external beam radiotherapy as defined by the DEGRO Working Group Radiosurgery and Stereotactic Radiotherapy and the DGMP Working Group for Physics and Technology in Stereotactic Radiotherapy. The covered aspects of this review are 1) imaging for target volume definition, 2) patient positioning and target volume localization, 3) motion management, 4) collimation of the irradiation and beam directions, 5) dose calculation, 6) treatment unit accuracy, and 7) dedicated quality assurance measures. For each part, an expert review for current state-of-the-art techniques and their particular technological quality requirement to reach the necessary accuracy for stereotactic radiotherapy divided into intracranial stereotactic radiosurgery in one single fraction (SRS), intracranial fractionated stereotactic radiotherapy (FSRT), and extracranial stereotactic body radiotherapy (SBRT) is presented. All recommendations and suggestions for all mentioned aspects of stereotactic radiotherapy are formulated and related uncertainties and potential sources of error discussed. Additionally, further research and development needs in terms of insufficient data and unsolved problems for stereotactic radiotherapy are identified, which will serve as a basis for the future assignments of the DGMP Working Group for Physics and Technology in Stereotactic Radiotherapy. The review was group peer-reviewed, and consensus was obtained through multiple working group meetings.

**Electronic supplementary material:**

The online version of this article (10.1007/s00066-020-01583-2) contains supplementary material, which is available to authorized users.

## Introduction

The Working Group Radiosurgery and Stereotactic Radiotherapy of the German Society for Radiation Oncology (DEGRO) and the Working Group for Physics and Technology in Stereotactic Radiotherapy of the German Society for Medical Physics (DGMP) jointly published a consensus statement for the definition of and quality requirements for intracranial stereotactic radiosurgery in one single fraction (SRS), intracranial fractionated stereotactic radiotherapy (FSRT), and extracranial stereotactic body radiotherapy (SBRT) [1], further jointly denoted as stereotactic radiotherapy if not specifically addressed.

Stereotactic radiotherapy is defined as a method of percutaneous external beam radiotherapy, in which a clearly defined target volume is treated accurately with a high radiation dose in up to 12 fractions with locally curative intent. Importantly in this context, a risk-adapted adjustment of the fractionation and the total dose based on the volume and location of the target is essential. Additionally, technological quality requirements in terms of 1) imaging for target volume and organs-at-risk definition, 2) patient positioning and target volume localization, 3) management of periodic and non-periodic target motion, 4) collimation of the irradiation and beam direction, 5) dose calculation, 6) geometric and dosimetric treatment unit accuracy, and 7) dedicated quality assurance measures were presented in the DEGRO/DGMP consensus statement. Furthermore, process quality requirements in terms of standard operating procedures, interdisciplinary discussion, training, experience, documentation, and reporting for stereotactic radiotherapy were defined.

The present expert review provides the background and details for each of the technological quality requirements for stereotactic radiotherapy as described in the consensus statement [1], taking into account the current state-of-the-art clinical practice. Furthermore, this review discusses and explores further needs for research and development to overcome unsolved problems.

## Expert review

For the investigation of existing methods, results and open questions for each technological quality requirement for stereotactic radiotherapy, an expert review was performed and summarized. For the specific technological quality requirement “treatment unit accuracy”, a dedicated distributed literature search in PubMed/Medline for the past 6 years (2013–2018) for the keyword combinations (sbrt OR sabr OR srs OR srt OR stereotaxy OR (stereotactic AND (radiotherapy OR radiosurgery))) AND either a) end-to-end, b) E2E, c) (mechanical OR geometric) accuracy, d) dosimetric accuracy, e) quality assurance, f) accuracy guidelines, g) dosimetry audit, or h) credentialing was performed.

In order to avoid bias, a group peer review with 15 reviewers (see acknowledgments) was performed to reach initial agreement on this expert review. Additionally, this review was openly discussed at two working group meetings before and after the group peer review, each with more than 100 participants, and consensus was obtained for remaining critical questions via ballots.

## Technological quality requirements for stereotactic radiotherapy

The following technology quality requirements are specified additionally and/or are substantiated specifically for stereotactic radiotherapy and do not render other established guidelines for general radiation protection and radiotherapy obsolete. In the following sections, we first present the specific technological quality requirements as defined by the DEGRO/DGMP consensus statement [1] and then discuss the current state-of-the-art and its limitations based on the literature review.

### Imaging for target volume definition

#### Requirement:

*The target volume and all organs-at-risk are defined using organ-specific imaging modalities and standardized imaging protocols dedicated for stereotactic radiotherapy procedures. The use of secondary imaging requires accurate registration with the thin-slice planning computed tomography (CT).*

The volume definition of intracranial and extracranial stereotactic targets and the definition of organs at risk (OAR) require the acquisition of organ- and indication-specific planning image data. In general, high-quality, thin-slice (organ and lesion volume-specific with ≤1 mm intracranial and 2 mm extracranial slice thickness [2]) non-contrast enhanced CT with maximum possible in-plane resolution (organ and lesion volume-specific with ≤1 mm intracranial and ≤1.5 mm extracranial pixel edge length) is the primary planning imaging modality for target volume and OAR definition and dose calculation purposes. The selection of scan length should not only consider target structures and radiation-sensitive structures that might be affected by the treatment or be necessary for dose documentation, e.g., the whole lung for lung treatments, but also consider the treatment technique, e.g., an extension of the target region of 15 cm in superior and inferior directions for non-coplanar SBRT [2]. If unambiguous target definition is not possible on primary planning imaging data, secondary image data must be acquired and registered to the primary data to achieve maximum confidence in delineating the gross tumor/target volume (GTV) and to minimize procedural safety margins [3]. In the following section, general aspects related to the registration accuracy are summarized, followed by organ-specific paragraphs to review secondary planning imaging modalities.

Primary and secondary planning image data should be acquired with the patient in treatment position using well-defined, standardized imaging protocols dedicated for stereotactic radiotherapy procedures. Planning data from external imaging devices are a major source of displacement in the patient anatomy (deviating immobilization devices, tabletop, etc.), non-standardized image quality, and undocumented artifacts. Poor image contrast and image artifacts considerably hamper the clinician’s capability to perform and verify image registration, but also influence the accuracy of image registration and, if applicable, the auto-detection of implanted fiducial markers [4]. Secondary magnetic resonance imaging (MRI) data are preferably acquired in the axial plane and on the same day as the primary planning CT, and the acquisition of dedicated simulation MRI for radiotherapy planning should be considered [5]. Each MRI sequence should be corrected for geometric distortions in the intrinsic imaging processing method of the MRI scanner (with a 3D approach if possible). The receiver bandwidth should be set to the highest value possible that still delivers an acceptable signal-to-noise ratio, as the accuracy of the MRI images (especially in tissue transition zones) could be a concern for contouring and for simulation [3, 5].

Overall, accurate image registration depends on image acquisition settings, sufficient spatial resolution, and image quality. It also relies on the selected registration algorithms and user-defined parameters (e.g., region of interest, soft tissue versus bone registration, landmarks, registration cost function, etc.). Rigid registration algorithms are currently considered the standard approach in radiotherapy practice [4, 6]. In areas of low contrast in the primary planning CT (e.g., in the liver), fiducial markers implanted prior to treatment planning imaging could improve the registration accuracy. However, rigid registration algorithms are challenged by non-linear deformations mainly associated with variation of organ position, shape, and volume between the primary and secondary image acquisition. Some non-linear deformations can be eliminated by the use of individual sub-volumes to optimize the registration process or through different importance factors to selected anatomical regions. In general, deformable image registration may further improve rigid registration results and minimize subsequent safety margins.

Regardless of the method, image registration requires careful validation before clinical implementation, and case-by-case evaluation due to large variation in the complexity and robustness of the registration approaches is strongly recommended [4, 6–8]. Furthermore, the use of automated registration methods or auto-detection algorithms for implanted fiducials requires the evaluation of the system’s performance for various image acquisition settings (e.g., to identify image quality limits where the algorithm fails) to establish appropriate institutional imaging registration protocols [4, 6].

#### Intracranial

Since lesions or OAR in the brain are often not visible on non-contrast-enhanced CT, MRI, or in case the patient cannot undergo MRI, contrast-enhanced CT is the standard imaging modality to define target volumes and OAR for intracranial indications. MRI planning images should be generally acquired with a maximum slice thickness of 1–1.5 mm and minimum field strength of 1.5 T [3, 9–11]. Contrast-enhanced T1-weighted imaging is commonly used for brain metastases or uveal melanomas. Of note for brain metastases: significant target volume changes may occur if the time between planning imaging and treatment delivery exceeds 1 week or if new decongestant medication is administered after planning imaging [12, 13]. For tumor resection cavities, additional pre- and direct postoperative (≤48 h after surgery [14]) contrast-enhanced T1-weighted sequences are desirable. For non-enhancing brain tumors, T2-weighted and/or fluid-attenuated inversion recovery (FLAIR) sequences or positron-emission tomography (PET) are mainly used for target definition. However, specific circumstances of a wide range of benign and malignant brain tumors will require additional specific sequences or the use of PET.

Target volumes of vascular disorders in the brain such as arteriovenous malformations (AVM) are defined using stereoscopic 2D digital subtraction angiograms (DSA) in combination with registered CT angiograms and/or MRI angiograms. Volumetric contrast-enhanced thin-slice (max. 1–1.5 mm) MRI and/or high-resolution contrast-enhanced CT scans and/or cone-beam computed tomography (CBCT) angiography are considered alternative imaging modalities [3, 10, 15], but typically lack detailed hemodynamic information compared to DSA imaging.

#### Spine/bone

Target volumes in the spine or in extracranial bones and OAR are commonly defined using high-resolution CT and/or MRI and/or PET or in case of spinal AVM, using DSA. MRI and/or PET may allow for better definition of the tumor lesion within the bone and potential epidural disease extension whereas T2-weighted MRI may provide the highest resolution. Volumetric T1-/T2-weighted MRI sequences are generally rigidly registered locally to a thin-slice planning CT (≤1–2 mm slice thickness depending on target volume and location). The delineation of the spinal cord can be performed using either T2-weighted MRI or a CT myelogram in cases where the patient is unable to undergo MRI or if the spinal cord is not clearly visible on MRI, e.g., due to significant metal artefacts [3, 16–18].

#### Lung/mediastinum

CT is the standard imaging modality for target definition of lung tumors and for OAR contouring. The planning CT (≤2 mm slice thickness) for lung tumors should cover the entire ipsi- and contralateral lung. To improve target volume definition, functional imaging such as FDG-PET or intravenous contrast-enhanced CT data (e.g., for central tumors) may be used, desirably time-resolved [19–21]. In addition to GTV definition, the planning image data is also acquired to assess patient-specific target motion and deformation as well as to evaluate correlation consistency of tumor motion in relationship to any internal or external markers prior to treatment. The CT acquisition technique (e.g., 4D-CT, prospectively gated multiphase CT, breath-hold CT) has to be chosen according to the motion management strategy applied during treatment delivery, as defined in the “Motion management” section below [3, 16, 17, 19–23].

#### Liver/abdomen

Planning CT imaging (≤2 mm slice thickness) for abdominal tumors is identical to lung tumors, though it should be noted that organ motion in the abdomen can be significantly larger than in the lung [24] and be non-periodic due, e.g., to digestion [25]. However, non-contrast-enhanced CT is in the vast majority cases not sufficient for GTV definition of abdominal tumors (e.g., for liver or pancreatic tumors) [26, 27] and for OAR contouring (e.g., for vessels). Secondary high-contrast image data are acquired through breath-hold or time-resolved (4D) contrast-enhanced CT [28, 29] and/or through breath-hold MRI using T2- and multiple T1-weighted sequences dynamically during and/or 0–20 min after injection of intravenous contrast agents. If applicable, the MRI breath-hold phase should coincide with the breathing phase of primary planning CT used for contouring. However, the CT/MRI may underestimate the abdominal tumor extensions compared to the pathological specimens [30, 31]. Additionally, (4D)-PET-CT imaging may, depending on the tumor histology, improve target volume delineation in contrast to the surrounding edema and in the presence of organ motion/deformation [3, 16, 17, 25–27].

#### Prostate

Target and OAR delineation for primary prostate cancer for stereotactic radiotherapy is performed using high-resolution (≤1.5 mm slice thickness) CT and MRI. The scan length should ideally be extended by 15 cm superiorly and inferiorly beyond the prostate to cover the testicles, bladder, sigma, rectum, and lower small bowel [2, 32]. Combined planning CT/MRI data reduce delineation variability compared to CT data alone. Additionally, prostate definition based on standard T1/T2 MRT sequences results in a smaller CTV (e.g., in the area of prostate apex and anterior rectal wall) and thus provides more effective sparing of the rectum and neurovascular bundle and additionally enables delineation and potentially sparing the urethra of high doses [3, 32–34]. Additionally, Prostate-specific membrane antigen(PSMA)-PET-CT could increase the definition accuracy of the intra-prostatic lesion if a simultaneous boost for SBRT is considered [35].

#### Discussion

Generally, non-contrast enhanced CT is the primary planning imaging modality, as the use of contrast agents or other imaging modalities could lead to inaccuracies in dose calculation and image registration during treatment. Many indications for stereotactic radiotherapy require high-resolution MRI or contrast-enhanced CT for accurate target and OAR delineation, for which in most clinical scenarios accurate registration to the primary planning CT is required [3, 6, 9–11, 16–27]. However, quality requirements (e.g., for optimal contrast, artefact reduction, slice thickness, and registration) and quality assurance (e.g., methods, tools, and interval) of planning imaging devices are largely lacking for stereotactic radiotherapy. Furthermore, the detailed documentation in published manuscripts of important imaging parameters (e.g., imaging and registration modalities, protocols and processing methods, and recommended window settings) or even the methods for contouring of the OARs (e.g., imaging modalities and average CT vs. multiple phase CT for moving organs) are hardly found. This makes the crucial target and OAR delineation process especially difficult to compare among institutions. Additionally, the time delay between planning imaging and treatment plays an important role for brain metastases [12, 13]; yet, for other indications, it is largely unexplored, and in general this factor is largely unreported in the literature. Lastly, consensus for follow-up imaging including corresponding quality requirements (e.g., registration to the treatment plan) and methods for differentiating tumor recurrence vs. radiation reactions is largely lacking, despite already existing studies in the literature [36–38].

### Patient positioning and target volume localization

#### Requirement:

*Daily in-room image-guidance and online correction of target position errors using on-board CT, supplementary in-room CT or stereoscopic X‑ray is required.*

- *For SRS, an invasive fixation using a stereotactic head frame can be used alternatively to image guidance.*
- *For SRS and FSRT, non-invasive fixation of the patient’s head is combined with image guidance.*
- *For SBRT, image-guidance of the target itself (or a surrogate structure highly correlated with the target) is required. The optimal image-guidance strategy is dependent on the tumor site and location and needs to consider the following principles:*
  - *in cases of target motion relative to the bony anatomy, image-guidance requires volumetric imaging with or without implanted fiducial markers or electromagnetic transponders or requires stereoscopic X‑ray imaging of the target itself or of implanted fiducial markers as target surrogate.*
  - *in cases at risk of serial organs-at-risk motion into areas of critical radiation doses, volumetric image guidance is recommended.*

For treatment delivery, the patient has to be immobilized and positioned identically to the primary treatment planning imaging. The target volume itself or appropriate surrogates, if the target is not visible on setup verification imaging (e.g., bony anatomy for brain or spinal lesions [39] or implanted fiducial markers for abdominal or prostate lesions [3, 16, 17, 25, 26, 40, 41]) have to be localized in the treatment room directly before treatment. Because of known data on possible intrafractional target motion [42–45], we strongly recommend to define an institutional maximum time delay between setup verification image acquisition and start of treatment for all treatment sites, with a time delay as short as possible. To meet the accuracy requirements for stereotactic radiotherapy, this time delay should not exceed 5 min for most treatment sites [42–45].

For patient positioning and target volume localization, there currently exist no state-of-the-art setup devices besides invasive stereotactic head frames that are able to guarantee the needed positioning accuracy for stereotactic radiotherapy without image guidance [46, 47]. All image acquisitions for image-guided radiotherapy (IGRT) are performed inside the treatment room (“in-room”), either on board of the treatment device or integrated into the fixed treatment room coordinate system to guarantee a stable geometric relation between the imaging and treatment position. It has to be ensured that the patient does not move due to the change in treatment table position (e.g., between CT-on-rails acquisition and treatment). If the target volume is not visible on setup verification imaging, the geometric relation between the target volume and its surrogates has to be extracted from primary treatment planning imaging and has to be assumed constant between planning imaging and treatment. If the relation cannot be assumed constant, additional margins have to be applied.

#### Intracranial stereotactic radiosurgery and intracranial fractionated stereotactic radiotherapy

Patient positioning and target volume localization for intracranial stereotactic radiotherapy can be performed with invasive rigid head frames using stereotactic localizers for SRS [48–50] or with image guidance for both SRS and FSRT [3, 9, 10]. FSRT with stereotactic frames requires multiple invasive frame fixations and fraction-specific treatment planning and should be avoided. Furthermore, targeting errors due to frame slippage of more than 2 mm are possible [51] and therefore image guidance should be performed, if available. Image guidance can be performed using stereoscopic X‑ray imaging [52, 53], in-room CT, or on-board CT imaging [54–56]. Because of higher mobility of the skull inside thermoplastic masks compared to invasive frame immobilization [57], intrafraction head motion monitoring and delivery adaptation in frameless SRS/FSRT is required in order to minimize the planning target volume (PTV) margins [9, 42, 58]. Methods for intrafractional motion monitoring can be found in section “Motion management”.

#### Extracranial stereotactic body radiotherapy

The intracranial image-guidance methods can be used for extracranial target volumes as well [19–21, 25–27, 44, 59]. For additional information on surrounding critical structures, volumetric imaging is preferred over stereoscopic imaging [60]. For some treatment sites, additional methods and techniques are available which can assist patient positioning and target volume localization. However, those methods are not yet able to replace the state-of-the-art X‑ray methods due to a lack of absolute target localization accuracy (e.g., ultrasound [61], surface scanning [62]) or a lack of clinical data for stereotactic treatments (electromagnetic tracking [63]). For targets moving with respiration or digestion, patient positioning and motion management strategies are discussed in the “Motion management” section below.

#### Discussion

There exist a large range of systems and methods for accurate setup imaging for stereotactic radiotherapy in clinical practice [48–63]. However, the question of which surrogates are appropriate for targets that are not visible on setup imaging [64–66] is beyond the scope of this review and needs further investigation. As an example, implanted fiducial markers may not always have a fixed inter- and intrafractional geometry with the actual target, and the number of fiducials (i.e., for rotation tracking), the geometric arrangement, and the distance to the target strongly influence treatment accuracy [67–70]. Additionally, special hybrid treatment devices enabling MR image guidance for direct localization of soft tissue targets [71] are emerging. Nevertheless, the current quality requirement as described above explicitly excludes MRI-guided stereotactic treatments as a standard, as this technique is considered to be under investigation with first available geometric and clinical data for MR-guided SBRT [72–74], but without long-term follow-up. Particularly for MR-guided intracranial treatments is there currently a lack of clinical experience [75].

### Motion management

#### Requirement:

*Systematic assessment and consistent consideration of periodic and non-periodic target motion during*

- *imaging for treatment planning;*
- *target volume definition;*
- *beam-delivery technique planning;*
- *dose simulation;*
- *target volume localization & repositioning; and*
- *dose application*

*using a time-resolved motion management strategy is required. Compensation of breathing-induced uncertainties can be performed using breath-hold technique, free-breathing with gated beam delivery, free-breathing with continuous beam delivery using the internal target volume (ITV) or mid-ventilation concept and free-breathing with dynamic tumor tracking.*

Intrafractional target motion describes any motion that occurs during the delivery of a radiation therapy treatment fraction and can be either periodic (breathing and/or heartbeat induced, relevant for, e.g., lung and upper abdominal lesions such as liver, pancreas, kidney, and adrenal glands) [24, 76, 77] or non-periodic (mainly induced by spontaneous patient or target motion or from digestion, relevant for, e.g., uveal, spine, intracranial, or prostate lesions) [43, 44, 57], which can induce systematic (e.g., baseline drifts) or random treatment errors [3, 4, 19, 20, 24–26, 45, 46, 58, 68, 72, 78–81].

Any form of motion management tries to minimize the relative motion between the target volume and the treatment beam. Besides patient and/or target immobilization and specific diet protocols to reduce non-periodic motion, two general strategies to actively manage intrafractional target motion during dose application can be applied: tracking and gating [3, 82–84]. All of these techniques require continuous or frequent position detection (motion monitoring) of the target or a surrogate during dose delivery.

1. Tracking is defined as the pursuit of the target with the beam, by automatically moving either the patient or the beam corresponding to the motion-monitoring signal. Implemented clinical systems for beam tracking are robotic-based [53] or gimbaled-based [85]. Under clinical investigation are systems using multileaf collimator (MLC) tracking [86, 87] or patient couch tracking [88]. There are three forms of tracking: free-breathing periodic motion tracking (e.g., for lung and liver targets) [89, 90] and non-periodic motion tracking (e.g., for intracranial, spinal, and prostate targets), where the target volume position is assumed to be constant between the updates of the motion-monitoring signal [44, 89, 91] or for some target volumes (e.g., in the pancreas) a combination of both tracking techniques may be required [92].
2. Gating is defined as the triggering of the beam-on/off status via a continuous motion-monitoring signal. The beam is turned on if the signal is inside a gating window, defined during treatment imaging and planning, and is turned off once the signal exits this gating window. There are three forms of gating: free-breathing periodic motion gating (e.g., based on a specific breathing phase or amplitude for lung and liver targets [93–95]) and non-periodic motion gating (e.g., based on breath-hold under deep inspiration or expiration for lung and liver targets [96, 97] or based on spontaneous motion in intracranial, uveal, spinal, and prostate targets [98–100]) or a combination of both periodic and non-periodic motion gating [72].

If no active motion management technique is available for targets with non-periodic motion, additional safety margins should be considered. For targets with small periodic motion (≤5 mm), the internal target volume (ITV) or mid-ventilation (MidV) approach may be used instead of an active motion management technique. For the treatment of targets with larger periodic motion (>5 mm), an ITV/MidV approach is the minimum requirement if no active motion management technique is available or feasible [3, 82]. The ITV/MidV approach is a passive motion management technique that integrates time-resolved image information (internal motion) into the target volume by unifying all GTVs (CTVs) from all breathing phase CTs to the ITV, or for some cases by using a maximum intensity projection CT dataset derived from the 4D-CT [101]. The MidV concept uses the CT of the breathing phase nearest to the time-weighted mean position of the GTV (CTV) and integrates the peak-to-peak amplitude in all three directions into an anisotropic PTV margin [102]. Thereby, the irradiated volume is enlarged such that the prescribed dose is sufficiently delivered to the target during treatment, while the MidV approach often leads to smaller irradiated volumes than the ITV approach [102]. To reduce the irradiated volume, it can be beneficial to reduce the motion amplitude, e.g., through abdominal compression or active patient breathing training [102]. The ITV/MidV approach performed under intrafractional verification of the target or surrogate motion can be considered as gating with a full-amplitude gating window. If for the ITV/MidV approach intrafractional verification of the target or surrogate motion cannot be performed, additional uncertainty margins should be considered.

Regardless of the motion management strategy, the coaching of breathing patterns (i.e., teaching the patient how to breath) and the training of breathing (i.e., repeating the taught breathing patterns) prior to and the audible and/or visual feedback of breathing during each of the treatment steps for stereotactic radiotherapy can be crucial to reduce treatment time, uncertainties, and errors [4, 103–106]. However, caution is strongly advised, as focused breathing may also lead to increased tumor motion and the feasibility and usefulness of coaching, training, and feedback should always be evaluated on a case-by-case basis.

The choice for one of these motion management strategies defines the necessary requirements for all steps in the treatment chain as described in the following.

#### Imaging for treatment planning

A free-breathing static CT of the lung and upper abdomen suffers from motion artifacts like blurring of structures and multiple representations of organ boundaries. It lacks valid time-resolved information about target position, size, and shape. To overcome these problems, respiration-correlated 4D-CT, prospectively gated multiphase CT, or a combination of inspiration and expiration breath-hold CT can be acquired [82]. The reconstruction of 4D-CT data is most frequently performed with a phase-based sorting algorithm and up to 10 respiration phases. However, phase-based sorting is more affected by breathing irregularity-related motion artefacts compared to amplitude-based sorting [3, 20–22].

The use of time-resolved imaging allows for an assessment of target motion and deformation as well as consistency validation of the relationship between the target and any surrogate used during dose delivery, regardless of the motion management strategy. For targets with non-periodic motion, a static CT should be used. Regardless of the target location and the motion management strategy, special attention to patient preparation (e.g., positioning, organ filling, and breathing pattern) has to be paid in order to reproduce the internal situation of patient anatomy at the time of planning imaging during treatment.

#### Target volume definition

Uncertainties in target position and shape during treatment have to be integrated into the target volume definition. Using an ITV/MidV approach, the target representation from all available image data (e.g., from 4D CT) can be integrated therein as described above, while the ITV needs an additional PTV margin, which is included in the MidV approach. For all motion management techniques, the PTV margin has to be enlarged such that all uncertainties from motion representation in planning imaging and all uncertainties related to the particular motion management strategy during treatment previously not considered are covered [89, 107]. Examples of motion management uncertainties arise from uncompensated residual target motion, rotation, and deformation [81, 108, 109], differential target and surrogate motion (e.g., of target and implanted fiducials or diaphragm [66]), target–surrogate correlation modeling [80], motion monitoring and correlation model update frequency [110], and motion prediction modeling due to latency times of the motion tracking systems [111, 112]. Typically for stereotactic radiotherapy, PTV margins range from 0–2 mm for intracranial/spinal targets to 3–5 mm for moving extracranial targets.

#### Beam-delivery technique planning

Beam technique factors (e.g., beam modulation, MLC leaf travel) may be considered when treating moving targets. Especially in some case scenarios with a low number of fractions (e.g., 1–3) in combination with high dose rates (e.g., when using flatting filter-free beams) such interplay effects between target motion and moving parts of the delivery system could be significant and unpredictable [113, 114]. However, averaging effects for higher fractionated stereotactic radiotherapy (e.g., 5 or more) generally result in a pure Gaussian blurring of the dose distribution that can be compensated by appropriate margins [115–117]. The simplest method to prevent interplay effects can be realized by avoiding modulated treatment sequences altogether.

#### Dose simulation

The dose delivery process is a time-dependent procedure, but typically modeled as a static procedure with according uncertainty margins in treatment planning. There are several emerging approaches to overcome the differences between static treatment planning and dose delivery under target motion (e.g., 4D dose calculation [118–121], 4D dose optimization [122, 123], and robust treatment planning [124]). These techniques usually assume reproducibility of the target motion at the time of imaging during treatment, which has to be verified during treatment to evaluate the reliability of the dose simulation.

#### Target volume localization & repositioning

For dose delivery, the assumptions made for target volume definition and treatment planning have to be fulfilled. Therefore, the target volume itself has to be localized and positioned before each fraction using the methods defined in “Patient positioning and target volume localization” above, adapted to the used motion management strategy. Any in-room imaging for target volume localization has to be accurately registered to the primary treatment planning imaging data. For periodically moving targets, stereoscopic X‑ray imaging requires short acquisition times. Furthermore, breathing phase information for in-room imaging is required (e.g., breath-hold position for on-board or supplementary in-room imaging, or 4D-CBCT [59, 125]). An exception to that is 3D-CBCT under free breathing. Here, the 3D-CBCT can be used for ITV verification due to the intrinsic averaging effects of the CBCT image acquisition [126]. The target volume localization based on the methods as described has to be performed repeatedly during treatment under consideration of the additionally applied safety margin. If during repeated target volume localization deviations from the treatment planning assumptions are detected that are not covered by the additional safety margin as described above, patient or beam repositioning or even plan adaptation is required.

#### Dose delivery

For all motion management strategies, monitoring of target motion during treatment, taking into consideration the applied safety margins, is required. Ideally, this may be performed in real time and using non-ionizing volumetric imaging [71, 127] (which is currently only available at a few centers). For non-periodic target motion, deviations from reference detected by continuous motion-monitoring systems may trigger renewed target volume localization and, if necessary, patient repositioning [51, 52, 128]. If for periodic target motion continuous motion monitoring during treatment is not feasible (e.g., due to target visibility or imaging dose constraints), accurate correlation modeling between the target and external surrogate signals (e.g., surface or artificial marker [53, 62, 85, 129], spirometry [130], etc.) or verification of the correlation model obtained during planning imaging is strongly advised for stereotactic radiotherapy. If the correlation model cannot be built or verified (not recommended), larger additional uncertainty margins must be considered. Correlation models have to be repeatedly verified during treatment based on the fraction duration and updated if necessary [131]. The motion management strategy must be adapted to the fraction duration, which, in turn, should be kept as short as possible for most applications (e.g., by using flattening filter-free beams with high dose rates).

#### Discussion

A large variety of technologies and methods for periodic and non-periodic motion compensation for all aspects of the stereotactic radiotherapy treatment chain has been presented [82–132]. However, some of the techniques lack standardized validation methods, mainly due to repeatability during treatment (e.g., advanced 4D motion modeling and 4D dose calculation or automatic registration of intrafractional images [6]). A similar related issue concerns interfractional changes in patient anatomy and daily adaptive re-planning using newly available high-contrast volumetric imaging (e.g., MRI). Respective workflows are under investigation and no general recommendations can be given at the present time. Furthermore, we want to emphasize that using an ITV/MidV concept for intrafractional breathing motion compensation without verification and monitoring of the actual target motion patterns before and during treatment cannot be considered a best practice for stereotactic radiotherapy of strongly moving targets. Several publications reported significant changes in breathing patterns and absolute target positions [133–135], which is particularly relevant when treating in very few fractions (i.e., 1–3). Future directions for motion management strategies for stereotactic radiotherapy will comprise continuous volumetric imaging during dose delivery [71, 72, 136] as well as online adaptation to possible changes in patient anatomy and motion patterns of the target volume. In the end, only the assessment of the in vivo tissue response will guide us towards the needed technical and biological treatment accuracy for stereotactic radiotherapy of moving targets [37].

### Collimation of the irradiation and beam directions

#### Requirement:

*For the respective treatment modalities, collimation and beam direction requires the following characteristics:*

- *SRS with multileaf collimator (MLC) with leaf width ≤5 mm or cylindrical collimators of equivalent size, both at normal treatment distance, and used with systems allowing non-coplanar beam directions.*
- *FSRT with MLC with leaf width ≤6.5 mm or cylindrical collimators of equivalent size, both at normal treatment distance.*
- *SBRT with MLC with leaf width <10 mm or cylindrical collimators of equivalent size, both at normal treatment distance.*

*However, for FSRT or SBRT close to radiation-sensitive critical structures the same collimation and beam direction requirements as for SRS are recommended.*

#### Cylindrical collimators

The inherent precision of fixed circular collimators together with their mount can be assumed to be in the range of what is standard in mechanical engineering, that is, 0.1 mm and better. This translates into a high agreement between the mechanical and radiological isocenter [137]. It is obvious that for small targets, a small collimator is needed to achieve an adequate conformity of the dose distribution to the target. Usually the lower limit of what is provided by manufacturers today is 4–5 mm. It should be noted that the dose gradient is, in principle, steeper for smaller collimator openings as can be shown for stereotactic convergent beam irradiation [138]. However, for inverse-optimized fluence-modulated delivery techniques, the effect of the single collimator might be concealed. Based on their inherent mechanical precision, fixed collimators are generally suited for SRS/FSRT and SBRT, at least if the collimator diameters for the according treatment device allow for adaptation to the actual tumor shape and size.

#### Multileaf collimators

Based on sampling theory, a lower MLC leaf width limit of 1.5 to 1.8 mm (related to normal treatment distance), below which the dose distribution cannot be refined further, can be assumed for radiotherapy [139]. However, the vast majority of MLCs presently used for stereotactic radiotherapy have leaves that are much wider.

One planning study compared MLC leaf widths of 2.5 mm and 5 mm using both step-and-shoot intensity-modulated radiation therapy (IMRT) and intensity-modulated arc therapy (IMAT) techniques on two different phantoms mimicking small- and large-field head and neck targets [140]. The study found that for small fields and a small c‑shaped target around an organ at risk (OAR), improved conformity for the 2.5 mm MLC was observed with a lower maximum for the OAR together with lower peripheral doses. This advantage was more pronounced for IMAT. In contrast, for so-called normal sized head and neck targets, the study did not find dosimetric benefits by using the 2.5 mm instead of the 5 mm MLC. Additionally, a study comparing a 3 mm MLC leaf width with a 5 mm MLC found that equivalent coverage with both MLCs can be achieved, whereas there was a statistically significant better conformity for the 3 mm MLC [141]. However, with both MLCs, the clinical predefined dose criteria (e.g., RTOG dose limits) could be fulfilled for all cases.

In another planning study on IMRT for prostate targets, the influence of the leaf width using three different MLCs with 2.5, 5, and 10 mm leaf width was investigated [142]. The study found significant improvements in dose coverage for the 2.5 and 5 mm MLCs in relation to the 10 mm leaf width, but no significant gain when reducing the leaf width from 5 to 2.5 mm. Similar to this, a study comparing three different MLCs with leaf widths of 2.5, 4, and 5 mm for IMAT of spinal targets with volumes between 24 and 220 cm^3^ concluded that any of these leaf widths can be used for spinal SBRT [143]. Furthermore, a study on the difference between planning with an MLC leaf width of 2.5 vs. 5 mm with respect to different levels of plan complexity, namely 5- up to 17-field IMRT and IMAT with one or two arcs for the treatment of pituitary adenomas, demonstrated coverage and conformity improvement with the smaller leaves of about 2% for the 5‑field IMRT and only about 0.5% for the two-arc IMAT technique [144].

These studies might be exemplary to show that there is a notable benefit in reducing the MLC leaf width from 10 mm, but the benefit of using leaves smaller than 5 mm seems only marginal, maybe except for small or very irregularly formed targets. However, the differences between treatment platforms can be compensated when using more complex delivery techniques (e.g., [144, 145]), besides other influences on the comparison, such as calculation grid size. Part of this ambiguity is likely caused by the fact that MLC leaf width on the one hand is an easily defined quantity, but on the other hand, the mechanical size of the leaves is only one parameter among many in defining the actual geometrical resolution for an MLC.

According to sampling theory, the 20–80% beam penumbra divided by 1.7 equals the optimal sampling distance, which can be half the size of the leaf width (achievable, e.g., by a couch displacement of half the leaf width) [139]. Hence, the beam penumbra is crucial for the geometrical resolution of an MLC and the penumbra is mainly defined by the construction of the collimator head. Manufacturers place the MLC at different positions with respect to the diaphragm(s) and, by the same token, to the patient, which can lead to variations in the penumbra by a factor of about 1.5 ([146], the principal differences between manufacturers of newer MLCs are similar). In addition, ideas exist to reduce the leaf resolution and penumbra for the given mechanical setups, e.g., by using a so-called virtual isocenter, which adds to the complexity of defining the treatment quality solely based on the MLC leaf width [147]. Another example of a factor influencing the beam penumbra is the elongated elliptical shape of the beam focus with a relation of 1 to 2 for the axes using an achromatic 270° bending magnet system [148]. From that, a dependence of the beam penumbra on the diaphragm angle would follow.

The importance of the beam focus size as a crucial beam parameter is widely stressed in connection with small field dosimetry (e.g., [3]). However, measurement or simulations are cumbersome and, to our knowledge, are nowhere included in routine quality assurance or beam commissioning. This leads to the question of how these parameters are correctly implemented into the treatment planning system at all, since in many cases the baseline data, e.g., beam profiles, consist only of measurements of the fixed jaws.

These considerations illustrate that a certain leaf width alone as a requirement to define treatment quality might not be meaningful. However, for a simple definition based on the literature, we can determine that an MLC leaf width of below 10 mm is mandatory for stereotactic radiotherapy and that a leaf width of about 5 mm or less is recommended.

#### Helical and coplanar-only radiotherapy

Helical radiotherapy requires special consideration, as the MLC leaf width might be larger compared to the gantry-based MLC systems used for SRS and FSRT (i.e., 6.25 mm) but the delivery technique is different (helical) and it is in fact claimed to be used clinically for SRS and FSRT (e.g., [149–154]). However, in connection with helical treatment, especially for SRS, the inherent coplanarity of this modality (and for others, too) might be an additional disadvantage [155–157]. When compared with other stereotactic radiotherapy treatment platforms, helical radiotherapy performed worse in some of the studies [158, 159], though not in all cases. In a planning study comparing forward-planned stereotactic conformal radiation therapy (SCRT), IMRT, and helical radiotherapy, the results demonstrated that helical radiotherapy was superior to SCRT, including a better hippocampal sparing for close target lesions [159]. Additionally, another study claimed that sparing the hippocampus was feasible with four treatment platforms, including one for helical radiotherapy [160]. Furthermore, in one study, helical radiotherapy was even favored over cone-based gantry-based linear accelerators in cases where OAR prevented the use of non-coplanar beams [158]. Likewise, another study could not show superiority (or inferiority) of helical radiotherapy over gantry-based systems for arteriovenous malformations in general, but found advantages for the former for targets with special geometry [161].

From a more theoretical point of view, one could apply the argument of the sampling theory regarding optimal leaf resolution especially to helical radiotherapy, because it is rather sampling and not leaf width which defines the resolution of an MLC [139]. The continuous couch movement, inherent to helical treatment, produces a sample width which is smaller than the field width parallel to the couch motion direction (minimal 1 cm) and the perpendicular leaf width itself, and thus should produce a better resolution than could be expected from mechanical size alone. In conclusion, there seems to be sufficient physical rationale to not exclude helical therapy from being used for FSRT. However, for very small targets and situations where non-coplanar beams might be of advantage, the use of helical radiotherapy is not advised [155–157].

#### Discussion

We found that the MLC leaf width may be a simple measure for assessment of the geometrical resolution of treatment platforms and hence serve as a quality requirement for stereotactic radiotherapy based on the literature [139–148]. However, looking deeper into the treatment plan quality, the MLC leaf width does not directly represent the minimal possible field size, which is, together with the beam penumbra and its origins (e.g., beam spot size), also an important parameter to define the geometrical resolution [162]. These influences should be further investigated, especially when looking at SRS and FSRT using very small MLC or circular collimator fields with inherent dosimetric inaccuracies, e.g., due to shielding of the primary source with the collimator. For very small fields formed by a dynamic aperture (MLC or, e.g., CyberKnife IRIS collimator [Accuray Inc, Sunnyvale, USA]), the accuracy and reproducibility of leaf (or aperture) positioning should also be considered in an analysis of dosimetric reliability. Another aspect of treatment plan quality is the simple fact that the hardware alone does not make a good treatment plan, which is always a combination of conformity to the target volume, steep dose gradients in the healthy tissue, and the technical applicability under realistic conditions. Treatment planning techniques (e.g., coplanar vs. non-coplanar techniques including table angle selection [157] and number of fields or beam energy) are strongly method dependent and significantly influence the treatment plan quality [155, 156, 163–166]. While this has been investigated for numerous indications and well-described methods exist to improve treatment planning [167], center or even user credentialing for stereotactic radiotherapy is still lacking in Germany.

### Dose calculation

#### Requirement:

*For stereotactic radiotherapy in areas with large density inhomogeneities the use of a dose calculation algorithm that takes into account lateral electron transport to correct for density inhomogeneities is required. The maximum grid size for dose calculation should be 1–2 mm according to the target lesion dimensions and the image resolution for target definition.*

Primarily, the dose calculation for stereotactic radiotherapy applications is performed on the primary treatment planning CT and the accuracy of a dose calculation algorithm is strongly influenced by the underlying physics modelling the transport of secondary particles (i.e., scattered photons and electrons). While this problem is less relevant for homogeneous situations and field sizes larger than about 3 × 3 cm^2^ (or associated to the range of electrons), it is crucial in areas with high density differences (e.g., in thoracic or head and neck regions) and for small field sizes especially when combined with high-energy treatment beams. The range of suitable beam energies is considered to be ≤10 MV due to the reduced range of secondary electrons, the reduced transmission through the beam defining system, and the reduced neutron production when compared with beam energies larger than 10 MV [3]. It is important to understand that it is mainly the management of the lateral spread of scattered particles that determines the accuracy level of a dose calculation algorithm. This is the background to distinguish between type‑A algorithms, which model only the primary particle transport correctly and in which the approximation of the secondary particle transport is generally based on equivalent path length scaling, and type‑B algorithms, which include more sophisticated models for the management of secondary particles [3]. Examples of type‑A algorithms are ray-trace algorithms, pencil beam algorithms, or fast Fourier convolution algorithms, which all ignore changes in lateral electron transport. Convolution/superposition algorithms as well as collapsed cone convolution algorithms, which take changes in lateral electron transport into account, are examples of type‑B algorithms. The anisotropic analytical algorithm (AAA) is considered an intermediate type-A/type‑B algorithm. The more advanced type‑B algorithms sometimes also referred to as type‑C algorithms, which explicitly consider the lateral particle transport, include Monte Carlo algorithms as well as solver of the Boltzmann transport equation [3, 168].

For radiotherapy applications using photon beams including stereotactic techniques, the dose calculation algorithm determines the absorbed dose within a volumetric element (dose scoring voxel) of a given CT dataset representing the patient’s characteristics. The resulting accuracy of the estimated dose distributions for the different dose calculation algorithm depends on the specific situation considered such as field size, photon beam energy, dose scoring voxel size, and tissue/density heterogeneity (i.e., treatment site specific), but also on the implementation of the algorithm itself. In addition, the accuracy depends on the accuracy of the beam model used by the dose calculation algorithm along with a careful commissioning and validation, especially with the focus of the foreseen clinical application [3]. This may include additional tasks like verification of output factors for very small field sizes and the consideration of appropriate dose calculation algorithm quality assurance procedures following updates or upgrades. Furthermore, it is important to understand not only the approximations used in the dose calculation model, but also its implementation in order to anticipate potential shortcomings for dedicated applications (e.g., high resolution of the fluence is more important for small than for large radiation fields).

Typically, the accuracy for small field sizes and large energies is reduced in tissues with densities substantially different from water (e.g., lung [169, 170]). In terms of dose scoring improved accuracy is reported, when smaller voxel dimensions are used [171]. For stereotactic applications, an isotropic grid size of smaller than or equal to 2 mm is considered appropriate [2]; however, for very small targets, dose grid resolution of 1 mm or better may be needed [172]. Regardless of the dose grid resolution and the dose calculation algorithms used, the resolution of the primary planning imaging on which the dose calculation is performed should always be equal to or better than the dose grid resolution. Many studies have been performed to investigate the level of accuracy for different dose calculation algorithms involving small field sizes and tissue inhomogeneities: in summary, type‑A dose calculation algorithms lead to dose overestimation in target volumes located in low-density tissue when compared with type‑B algorithms or with detailed measurements [3, 173]. The approximations used in these algorithms lead to inaccurate dose distributions when low-density tissue is involved and thus are not appropriate for stereotactic treatment planning. Studies showed that intermediate type-A/type‑B algorithms, i.e., including the AAA algorithm, provided acceptable estimation of dose distributions for stereotactic applications if no low-density tissue is present, but they are of limited accuracy in the context of lung SBRT [168, 174–177]. Investigations of the performance of type‑B algorithms demonstrate the suitability of these algorithms for stereotactic radiotherapy purposes [2, 173, 178, 179]. Finally, studies showed that advanced type‑B (or type-C) algorithms accurately calculate dose for small fields in heterogeneous tissue and are best suited for stereotactic radiotherapy treatment planning, as recommended by the International Commission on Radiation Units and Measurements (ICRU) report 91 [3].

#### Discussion

It is largely agreed upon that dose calculation should be as accurate as clinically feasible and density inhomogeneities can be addressed with modern dose calculation algorithms [2, 168–179]. However, another aspect is the influence of the dose calculation algorithm on the actual prescription (e.g., based on different dose calculation or PTV coverage based vs. GTV mean dose based [3]) in clinical protocols. Given the experience gathered for a specific protocol, it is not trivial to transfer such a protocol and clinical experience to another, typically more advanced, dose calculation algorithm, since in general the difference in the estimated dose distribution due to different dose calculation algorithms is not a simple scaling factor [180–182]. Additionally, no consensus has currently been reached for the actual primary planning CT for dose calculation for targets with period motion (e.g., mean-intensity-projection, specific breathing phase with or without density overwrite, etc.) [3]. Hence, harmonization in dose prescription based on similar accurate dose calculation algorithms is still largely lacking. Furthermore, not only the target volume has to be considered, but the differences that might occur in organs at risk are also important [183, 184].

Furthermore, MRI is often used due to the high soft tissue contrast and the MR images are then registered to the CT dataset in treatment planning [185]. In order to overcome image registration errors, the use of MR-only treatment planning is of increasing interest [186]. While MR-only planning is common practice for Gamma Knife treatments, a synthetic CT is typically generated based on the MR image dataset when using other treatment modalities. However, there are currently still limitations in the MR-only approach for stereotactic radiotherapy, which comprise dose calculation uncertainties due to errors in HU assignments and image distortions [186]. Currently, there is no consensus whether these errors counterbalance the benefits of an MR-only planning approach. Additionally, beam delivery under MR real-time guidance may necessitate incorporating the MRI-based dose distortions into dose calculation, especially in areas with density inhomogeneities. Thus, additional studies have to be performed on this topic.

### Treatment unit accuracy

#### Requirement:

*A geometric accuracy with three-dimensional spatial dose placement in system-specific end-to-end tests requires inaccuracies of at maximum:*

- *1 mm for SRS.*
- *1.25 mm for FSRT and SBRT in non-moving phantoms.*
- *1.5 mm for SBRT in moving phantoms.*

*However, for FSRT and SBRT close to radiation-sensitive critical structures the same geometric accuracy requirement as for SRS is recommended.*

*A dosimetric accuracy with point-based plan-to-measurement differences of maximum 3% within a target volume of more than or equal to 2 cc, measured in system-specific end-to-end or delivery-quality-assurance tests with homogeneous phantoms, is required. For target volumes smaller than 2 cc, the uncertainties of the measurement may be larger than the desired dosimetric accuracy.*

The dedicated distributed literature search in PubMed/Medline revealed a total of 462 publications for the keywords used. After conference abstracts removal and headline screening by experts in the field, a total of 53 publications remained for further screening. Due to the nature of a distributed literature search, the results were then combined and the duplicates were removed. After final abstract screening and after adding well-known guidelines for stereotactic radiotherapy not listed in PubMed/Medline, a total of 35 publications and guidelines remained for full data extraction. The complete data extracted for the publications and guidelines can be found in Table 1 in the supplementary material (Online Resource 1).

Concerning international guidelines, the IAEA technical report series 483 advised a geometric accuracy of 2 mm and a dosimetric accuracy of 3–10% in system-specific end-to-end tests [154]; however, this advice is for radiotherapy in general and not specifically tailored to stereotactic radiotherapy. The ESTRO/ACROP guideline for lung SBRT on the other hand advised a geometric and dosimetric accuracy of 0.5–4 mm (median 1.25 mm) and 2–5% (median 3%), respectively [20]. Other notable international guidelines, including the AAPM-RSS Guideline 9a for SRS-SBRT, recommended a geometric accuracy of 1 mm for static and 1.5 mm for moving targets with 5% dosimetry accuracy [172]. This is in line with previously published system-specific AAPM TG reports [187]. On the other hand, the recent COMP reports for the same system advised on a geometric accuracy of 0.75 mm for static and 1.0 mm for moving targets based on recent developments in the system’s architecture [188]. Interestingly, no guideline reported specifically on the overall phantom test uncertainty, but of course the phantom test uncertainty itself plays an important role in the measured overall accuracy of the stereotactic treatment system as well. One example is the lack of recommendations on the necessary slice thickness for the phantom CT scan used for the end-to-end tests and we strongly recommend achieving the highest possible resolution, at least not worse than the resolution used for patient scans.

Furthermore, there are numerous guidelines which only present overviews of the published literature without giving clear recommendations, including the ICRU report 91 [3] and the AAPM TG 101 report [2]. The geometric accuracy in these reports ranges from 0.28 ± 0.36 mm to 1.5 ± 0.7 mm depending on system, test scenario, and measurement device. Adding recent literature to these guidelines, one can safely assume on one hand that well-calibrated dedicated stereotactic radiotherapy equipment can reach the required level of mechanical precision of 1 mm in system-specific static end-to-end tests for SRS [189–197]. Additionally, a geometric accuracy of ≤1 mm is clearly necessary for SRS in order to keep the overall PTV margin ≤2 mm when also considering patient motion during treatment before side effects increase significantly for the high SRS doses [9]. At this point, it should be noted that a PTV margin of 0 mm, which is often used in SRS, is geometrically infeasible with the current treatment units under consideration of patient and target motion. Hence, the use of a 0 mm PTV margin may be derived from clinical decisions and potentially be compensated by higher prescription doses.

When looking at non-continuous volumetric imaging alone (i.e., CBCT), there are a number of reports of end-to-end tests exceeding the limits of 1 mm [3, 198–201], even though the recent COMP report on CBCT advised achieving a geometric accuracy of <1 mm [201]. Hence, the accuracy of CBCT alone may not necessarily be sufficient for SRS, especially when further considering patient motion during treatment (see the “Patient positioning and target volume localization” and “Motion management” sections above). On the other hand, for FSRT and SBRT in non-moving organs, the required geometric precision of 1.25 mm should be achievable with well-calibrated CBCT systems, which then may be sufficient to keep the overall treatment accuracy below 2–3 mm when considering patient motion during treatment [9]. This recommendation is also in line with guidelines and studies including helical therapy and recently introduced integrated MR-linac devices [3, 189, 190, 202, 203]. However, when looking at irregular moving targets for SBRT, the geometric accuracy of any system in current clinical use may quickly exceed the required limits of 1.5 mm in moving phantoms [172, 187, 204], and only careful motion modeling and compensation may allow the overall PTV margins to be kept below 3–5 mm when additionally considering patient motion and local target baseline shifts and deformation during treatment (see “Motion management” section).

Concerning the dosimetric accuracy of the treatment systems used for stereotactic radiotherapy, high-quality data arise mostly from clinical trial audits. In homogeneous phantoms, the dosimetric end-to-end or delivery-quality-assurance accuracy was found to be well below the required 3% limit [190, 197, 205–207]. However, when looking at moving targets and especially those targets surrounded by heterogeneous tissue, it becomes apparent that the 3% dosimetric accuracy cannot always be reached without type‑C dose calculation algorithms (see “Dose calculation” section) [205].

#### Discussion

All dedicated and well-tuned devices for stereotactic radiotherapy can fulfill the obligations for high geometric and dosimetric accuracy during end-to-end tests [2, 3, 9, 89, 172, 187–219]. However, when target motion comes into consideration, the acceptable limits may quickly be exceeded for certain scenarios. Furthermore, clear recommendations for the acceptance of end-to-end tests for moving targets in heterogeneous tissues are currently lacking. Furthermore, no data exist to support a recommendation on any system-specific end-to-end or delivery-quality-assurance test for small target volumes (i.e., ≤2 cc). Due to current measurement uncertainties for very small radiation fields, we cannot recommend any valid approach or solid reference at this time [148, 220]. This should be further explored. Additionaly, no clear recommendations on the method and the acceptance criteria of delivery-quality-assurance tests can be given, since the literature including international recommendations is largely heterogeneous and even contradictory to some degree.

### Dedicated quality assurance measures

#### Requirement:

*Dedicated quality assurance measures are required*:

- *Small field dosimetry for commissioning.*
- *System specific end-to-end testing for both static and moving target volumes.*
- *Regular check of the geometric and dosimetric accuracy according to system-specific guidelines.*
- *Day-to-day quality control of the consistency of the stereotactic frame and/or the image-guidance system isocenter with the treatment beam isocenter.*

Stereotactic radiotherapy makes use of small radiation fields due to the generally small targets treated [3]. Hence, it becomes necessary to use adequate measurement tools and compensate for measurement uncertainties in small fields specifically during system commissioning (e.g., using correction methods for output factor measurements for the smallest field sizes), but also for the whole quality assurance chain. For small field dosimetry, the IAEA has recently reported a comprehensive practical guideline [208] and the German Institute for Standardization (*Deutsches Institut für Normung* [DIN]) has published a new norm for the dosimetry of small photon beams [220], both which we consider mandatory state-of-the-art practice for stereotactic radiotherapy.

Further, for full commissioning of radiotherapy devices used for stereotactic radiotherapy, dedicated full treatment chain end-to-end tests are required [221]. In this regard, a full treatment chain end-to-end test phantom validation must include all treatment steps including primary and secondary imaging and its registration and subsequent target volume delineation (“Imaging for target volume definition”), dose calculation (“Dose calculation”), phantom positioning and target volume localization (“Patient positioning and target volume localization”), and target motion compensation (“Motion management”), and must fulfill the specified geometric and dosimetric accuracy (“Treatment unit accuracy”). Therefore, system-specific end-to-end tests both for static (i.e., cranial) and for moving (ideally for both periodically, e.g., lung and liver, and non-periodically moving, e.g. prostate) target volumes are required.

Once the stereotactic radiotherapy device is fully commissioned, regular checks of the geometric and dosimetric accuracy have to be performed in order to ensure the system’s integrity and to find system drifts and inaccuracies early before clinical consequences can arise. There are a wide range of system specific recommendations available which are considered mandatory state-of-the-art practice for each of the systems in description [172, 187, 188, 201, 209, 221, 222]. Parts of these recommendations are specific details on daily quality assurance which minimally requires verification of the consistency of the stereotactic frame and/or the image-guidance system with the treatment isocenter (which is a modern version of the so called Winston–Lutz test [223], normally reduced in number of inspected axes for a daily check).

#### Discussion

Recently, comprehensive small-field dosimetry and system-specific quality assurance guidelines have been published for stereotactic radiotherapy [172, 187, 188, 201, 208, 209, 220–222]. However, the translation from the old to the new methods has only just begun and the clinical implications are not yet fully understood (e.g., when correcting the output factors and hence changing the absolute dose for high-dose small-field SRS for trigeminal neuralgia). Another point is that contouring based on MRI as well as treatment planning are generally not part of the end-to-end test chain, though they clearly should be. Furthermore, generally only the simplest forms of end-to-end tests are performed, leaving many treatment scenarios unverified (e.g., non-isocentric and/or simultaneous multiple lesion treatment techniques and complex moving and/or deforming target volumes with dual periodic, i.e., respiratory and cardiac motion, and/or non-periodic motion). However, current phantoms in routine clinical practice may not even allow for such scenarios to be simulated. Additionally, the interval for repeating full treatment chain end-to-end and delivery-quality-assurance tests is not well agreed upon. Regardless, independent validation methods and external audits are also lacking specifically for stereotactic radiotherapy in Germany.

## Conclusion

The present expert review from the DGMP Working Group for Physics and Technology in Stereotactic Radiotherapy summarizes technological quality requirements for stereotactic treatments using photon external beam radiotherapy in the form of necessary minimum requirements often complemented by recommendations or suggestions for optimal quality. Additionally, open questions in each of the seven sections are identified. “Imaging for target volume definition” emphasizes the need for indication-adapted treatment planning imaging, gives information on the use of multiple secondary imaging datasets for many treatment sites, and complements this with statements on reliable image registration with the planning CT.

In “Treatment unit accuracy” the requirements for interfractional patient positioning and target volume localization based on daily IGRT depending on technique (SRS, FSRT, SBRT) and visibility and anatomic properties of the target volume and nearby critical structures are described. These requirements are extended to further methods supporting intrafractional target volume localization as illustrated in “Motion management.” Here, the whole treatment chain from treatment planning imaging to dose delivery and their specific requirements for consistent motion management is described. Thereby, the choice of the motion management technique is considered the most important first step, as the technique will have strong impact on each individual link in the treatment chain.

“Collimation of the irradiation and beam directions” summarizes the requirements on beam collimation and beam directions, with a detailed analysis of the influence of MLC leaf width and other hardware components on the treatment plan quality. Adding to that, in “Dose calculation,” the necessity of a dose calculation algorithm suited to the tissue density properties in the target region is emphasized and explained. This necessity is extended by a description of the influence of the dose grid resolution and beam energy on the dose calculation accuracy.

The requirements of the geometric and dosimetric treatment unit accuracy with static and moving homogeneous phantoms are given and discussed in “Treatment unit accuracy.” This section is supported by a detailed supplementary table containing published recommendations and data for various treatment systems. Lastly, “Dedicated quality assurance measures” describes the necessary quality assurance procedures to guarantee the needed technological quality requirements from commissioning to end-to-end testing for static and moving targets up to required daily quality checks.

## Caption Electronic Supplementary Material

Supplementary table 1: Table with published accuracy values for stereotactic treatment units. *n.s*. not specified.

## Abbreviations

AAA: Anisotropic analytical algorithm
AAPM: American association of physicists in medicine
ACROP: Advisory committee on radiation oncology practice of the ESTRO
AVM: Arteriovenous malformation
CBCT: Cone-beam computed tomography
COMP: Canadian organization of medical physicists
CT: Computed tomography
CTV: Clinical target volume
DEGRO: Deutsche Gesellschaft für Radioonkologie (German Society for Radiation Oncology)
DGMP: Deutsche Gesellschaft für Medzinische Physik (German Society for Medical Physics)
DIN: Deutsches Institut für Normung (German Institute for Standardization)
DSA: Digital subtraction angiogram
ESTRO: European Society for Radiotherapy and Oncology
FLAIR: Fluid-attenuated inversion recovery MRI sequence
FSRT: Intracranial fractionated stereotactic radiotherapy
GTV: Gross tumor volume
IAEA: International Atomic Energy Agency
ICRU: International Commission on Radiation Units and Measurements
IGRT: Image-guided radiotherapy
IMAT: Intensity-modulated arc therapy
IMRT: Intensity-modulated radiotherapy
ITV: Internal target volume
MidV: Mid-ventilation
MLC: Multileaf collimator
MRI: Magnetic resonance imaging
OAR: Organ at risk
PET: Positron-emission tomography
PSMA: Prostate-specific membrane antigen
PTV: Planning target volume
RSS: Radiosurgery Society
SBRT: Extracranial stereotactic body radiotherapy
SRS: Intracranial stereotactic radiosurgery
