# Supplementary material for: Technological quality requirements for stereotactic radiotherapy: Expert review group consensus from the DGMP Working Group for Physics and Technology in Stereotactic Radiotherapy
Source: Strahlenther Onkol. 2020 Mar 24;196(5):421–43. doi: 10.1007/s00066-020-01583-2 (PMC7182540; doi:10.1007/s00066-020-01583-2)
Supplement: Supplementary file 1 — Supplementary table 1: Table with published accuracy values for stereotactic treatment units. n.s. not specified. [file 66_2020_1583_MOESM1_ESM.pdf]

Strahlentherapie und Onkologie

Supplementary material to:
"Technological quality requirements for stereotactic radiotherapy
Expert review group consensus from the DGMP Working Group for Physics and Technology in Stereotactic Radiotherapy"

Daniela Schmitt*, Oliver Blanck, Tobias Gauer, Michael Fix, Thomas B. Brunner, Jens Fleckenstein, Britta Loutfi-Krauss, Peter Manser, Rene Werner, Maria-Lisa Wilhelm, Wolfgang W. Baus, Christos Moustakis

*Universitätsklinikum Heidelberg, Klinik für Radioonkologie und Strahlentherapie, Heidelberg, Deutschland und National Center for Radiation Research in Oncology (NCRO) und Heidelberger Institut für Radioonkologie (HIRO), Heidelberg, Deutschland
E-Mail: Daniela.Schmitt@med.uni-heidelberg.de

**Reference (Year Published)** (Sub-Reference, Year published)	**Mechanical Precision in End-To-End Test** (median/range or mean + standard deviation)	**Dosimetry Precision in End-To-End Test** (median/range or mean + standard deviation)	**Dosimetry Precision in Patient Specific Phantom Test** (median/range or mean + standard deviation)	**Report / Tested System**	**Measurement Tools**	**Volume Specific Recommendation**	**Comments**
IAEA Technical Reports Series 483 (2017) [4]	2mm	3–10%	5–10%	General Report	n.s.	n.s.	For EBRT only, SRS/SRT/ SBRT not specified
ESTRO / ACROP Guideline for Lung SBRT (2017) [20]	1.25mm (0.5–4 mm)	3% (2–5%)	No Consensus	Consensus Guideline	n.s.	n.s.	For lung SBRT only, small consensus, no procedural details
ICRU Report 91 (2017) [3]		n.s.	n.s.	Expert Guideline	n.s.	n.s.	References only, no recommendation for end-to-end accuracy
(Hacker, 2006)	≤ 1.5mm ± 0.7mm			ExacTrac			
(Jin, 2008)	0.56mm ± 0.7mm			ExacTrac			
(Verellen, 2003)	0.6mm ± 0.9mm			ExacTrac			
(Antypas, 2008)	0.56mm ± 0.7mm			CyberKnife			

(Desai, 2006)	0.8mm ± 0.05mm			CyberKnife			
(Kilby, 2010)	0.4mm ± 0.1mm<br>0.5mm ± 0.2mm<br>0.3mm ± 0.1mm			CyberKnife<br>- 6D Skull<br>- XSight Spine<br>- Fiducial			
(Hyde, 2012)	< 1.4mm and < 0.7°<br>(95% CI)			CBCT			
(Sharpe, 2006)	1–1.5mm (95% CI)			CBCT			
(Fenwick, 2004)	±1mm			TomoTherapy			
(Boswell, 2006)	1mm			TomoTherapy			
(Depuydt, 2011)	0.54mm ± 0.2mm			VERO			
AAPM-RSS Guideline 9.a (2017) [172]				Expert Guideline			To be performed annually/quarterly, no details provided
	1.0mm	5%	n.s.	C-Arm LINAC / TomoTherapy	n.s.	n.s.	
	1.0mm (static)<br>1.5mm (moving)	n.s.	n.s.	CyberKnife	n.s.	n.s.	
Dosimetry Audit in lung SBRT trials (2017) [206]			n.s.	Expert Report	Anthropomorphic lung phantom	n.s.	New and large data included, though no recommendation, Lung SBRT only
NRG LU002, BR001, BR002, LUSTRE OCOG (IROC, 2013)	n.s.	0.9%<br>1.8%<br>3.2%<br>3.8%		Acuros<br>Monte Carlo<br>Superposition<br>AAA	TLD, Film		
SARON, CORE (UK, 2017)	3mm (-6–7mm) 100%<br>5mm (-10–19mm) 50%<br>Isodose-to-Isodose	0.7% (-1.0–2.6%)<br>0.3% (-2.9–3.2%)<br>0.4 ± 1.4%<br>1.4 ± 3.4%		Conformal<br>IMAT	Alanine Pellets, Film		

AAPM TG 101 Report (2010) [2]		n.s.	n.s.	Report with Guideline		n.s.	References only, no recommendation
(Ryu, 2001)	1.0–1.2mm (RMS)			Stereo X-Ray	Hidden target		
(Verellen, 2003)	0.41 ± 0.92 mm			Stereo X-Ray	Hidden target		
(Verellen, 2003)	0.28 ± 0.36 mm			Stereo X-Ray	Fiducials		
(Yu, 2004)	0.68 ± 0.29 mm			Stereo X-Ray	Fiducials		
(Solberg, 2008)	1.10 ± 0.42 mm			Stereo X-Ray	Anthropomorphic phantom		
COMP Report Gamma Knife (2018) [209]	n.s.	1–5%/0.5 mm (Gamma, no specifics)	n.s.	Report with Guideline / Gamma Knife	Appropriate (1% with Ion-Chamber, 5% with Film	n.s.	No specific procedural details given
COMP Report CyberKnife (2018) [189]	0.75mm (static) 1mm (moving)	n.s.	Gamma with absolute dose and 90% pass rate: 5%/2mm (static) 5%/3mm (moving)	CyberKnife	Appropriate (≤10 mm collimator with Film)	≤10mm collimator split for measurement tools	For mechanical accuracy stricter (newer systems), but for DQA weaker recommendations as AAPM TG 135
COMP Report CBCT (2018) [202]	±1mm	n.s.	n.s.	CBCT	n.s.	n.s.	No specific procedural details given
AAPM TG 135 Report (2011) [188]	<0.9mm (static) <1.5mm (moving)	n.s.	Gamma with absolute dose and 90% pass rate: 2%/2mm (static) 3%/3mm (moving)	CyberKnife	n.s.	n.s.	No specific DQA procedural details given, for old systems only
Wen et al. (2018) [204]	1.0 ± 0.1 mm	n.s.	1.64 ± 1.90% (-3.50–0.79%) Gamma (no details) 96.87% (3%/1mm)	MR-Linac	Film	n.s.	Monocentric, prototype phantoms
Pantelis et al. (2018) [212]	Gel <0.66 mm 0.4 ± 0.18mm 0.55 ± 0.20 mm 0.40 ± 0.19mm	n.s.	n.s.	CyberKnife - 6D Skull - XSight Spine - Fiducial	Gel Film	n.s.	Monocentric, but comprehensive

Kron et al. (2018) [201]	< 3 mm (stationary)	0-2.6% difference between moving and stationary measurement	n.s.	CBCT	Film with motion platform	n.s.	Multicenter, lung only, limited evaluation quality
Kurosu et al. (2017) [217]	n.s.	n.s.	1.0 ± 0.9% (isocentric)<br>0.5 ± 1.4% (conformal, homogeneous dose)<br>4.1 ± 2.8% (conformal, inhomogeneous dose)	CyberKnife	Ion-Chamber	n.s.	Monocentric, point dose measurement error analysis based on dose gradient
Bellec et al. (2017) [218]	n.s.	n.s.	Gamma 3% relative dose and 1.5mm DTA >88% for error detection	CyberKnife	Film<br>Process-Control-Statistics	n.s.	Monocentric, large data set, process control
Calvo Ortega et al. (2017) [199]	1.4 ± 0.2mm<br>1.8mm (95% CI)	n.s.	n.s.	CBCT	Head Phantom<br>Film	n.s.	Monocentric,<br>SRS only
Colodro et al. (2017) [216]	n.s.	n.s.	Gamma 3%/1.5mm (absolute dose)<br>98.8% (95.8–100.0%)<br>99.3% (97.9–99.9%)<br>(relative dose)<br>80.7% (65.2–92.9%)<br>92.7% (87.5–97.3%)	CBCT	Ion-Chamber Array<br>Film	6.8-79.4ccm	Monocentric, lung SBRT only, gamma pass rate analysis
Dimitriadis et al. (2016) [200]	0.8mm (0.1-2.0mm)	n.s.	n.s.	All	n.s.	n.s.	Questionnaire, SRS only
Fürweger et al. (2016) [195]	0.38 ± 0.05mm	n.s.	n.s.	CyberKnife	Film	n.s.	Monocentric, small series
Calvo Ortega et al. (2016) [203]	-0.4 ± 0.6 mm LR<br>-0.1 ± 0.4 mm AP<br>0.2 ± 0.4 mm SI	n.s.	n.s.	CBCT	Head Phantom	n.s.	Monocentric,<br>SRS only
Wen et al. (2016) [211]	0.44–0.64mm (beam positioning)<br>0.27–0.61mm (gantry rotation)	n.s.	n.s.	Edge	Film	n.s.	Monocentric, prototype phantom, SRS/SRT only

Colvill et al. (2016) [89]	n.s.	n.s.	Gamma 2% absolute dose and 2mm DTA<br>Motion Compensation<br>98.4% (lung)<br>98.6% (prostate)<br>No Compensation<br>84.8% (lung)<br>82.7% (prostate)	C-Arm with<br>- MLC Tracking<br>- Couch Tracking<br>CyberKnife<br>Vero	Diode or Ion-Chamber Array or Film with motion platform and multiple given motion traces	7.2cc (lung)<br>55.3cc (prostate)	Multicenter benchmark for motion compensation systems, large variety in measurement systems, SBRT only
Sothmann et al. (2016) [205]	-1.0–0.7mm (LR)<br>-0.6–0.1mm (SI)<br>-0.8–0.2mm (LR)<br>-0.8–0.4mm (SI)	n.s.	1–2% (regular motion)<br>2–4% (complex motion)<br>1–2% (regular motion)<br>2–3% (complex motion)	CyberKnife<br>Vero	Ion-Chamber Array with motion platform and patient motion	30-180ccm (liver)	Benchmark and error classification for motion changes, SBRT only
Blanck et al. (2016) [197]	0.15mm (end-to-end)<br>0.97 (0.1–1.8mm)<br>(patient specific QA)	n.s.	Gamma 3% relative dose and 1mm DTA<br>95.8% (90.5–100%)	CyberKnife	Ion-Chamber Array	n.s.	Multicenter, geometric errors attributed to array
Sarkar et al. (2016) [208]	n.s.	-1.4–1%	Gamma 2% absolute dose and 2mm DTA<br>83.4%–98.0%	Frame<br>CBCT<br>Stereo X-Ray	Ion-Chamber<br>Film	n.s.	Phantom / technique comparison, small study, monocentric
Blanck et al. (2015) [196]	0.55mm (end-to-end)<br>0.45mm (0.2–0.9mm)<br>(patient specific QA)	n.s.	Gamma 3% relative dose and 1mm DTA<br>95.45% (81.3–100%)	CyberKnife	Film	n.s.	Multicenter, dosimetric errors attributed to film
Gallo et al. (2015) [207]	n.s.	1.5% (1.4-3.1%)<br>Gamma with absolute dose and 90% pass rate:<br>96.1–100% (3%/3mm)<br>93.3–100% (2%/2mm)<br>49.8–95.4% (1%/1mm)	n.s.	CBCT<br>Tomotherapie<br>CyberKnife<br>Vero	Ion-Chamber<br>Film<br>Dedicated Spine Phantom	n.s.	Multicenter, comprehensive, Spine SBRT only
Seravalli et al. (2015) [190]	Σ 0.49mm<br>σ 0.53mm	n.s.	n.s.	CBCT	Film	n.s.	Monocentric, but comprehensive, SRS only
Wen et al. (2015) [191]	0.66 ± 0.18mm<br>(drift over 3 months)	1.3 ± 0.9%	Gamma (no details)<br>99.4 ± 0.8% (3%/3mm)<br>93.0 ± 6.0% (3%/1mm)	Edge	Film<br>Ion-Chamber	n.s.	Monocentric, small test series

Swamy et al. (2015) [213]	n.s	n.s.	-2.5% (-4.6–-1.2%)<br>-1.6% (-5.1–3.0%)<br>-0.01% (-2.6–2.9%)	CBCT	CC13 Ion-Chamber<br>A14 Ion-Chamber<br>W1 Ion-Chamber	>10cc	Monocentric, small test series
Solberg et al. (2014) [194]	0.27mm LR<br>0.34mm AP<br>0.27 mm SI	n.s.	n.s.	Vero	Film	n.s.	Monocentric, commissioning only
Ma et al. (2014) [192]	0.03 ± 0.2 mm (2σ)	n.s.	n.s.	Gamma Knife	Film	n.s.	Monocentric, but large data, SRS only
Novotny et al. (2014) [193]	<0.2mm	n.s.	n.s.	Gamma Knife	Film<br>Diodes	n.s.	Monocentric, but long time data, SRS only
Mamalui-Hunter et al. (2013) [210]	n.s.	n.s.	2.3% (-5.7–4.9%)	Gamma Knife	Independent Dose Calculation	n.s.	Monocentric, no measurement, SRS only
Thomas et al. (2013) [214]	n.s.	n.s.	3D Gamma (no details)<br>99.9% (5%/3mm)<br>98.0% (3%/2mm)	Stereo X-Ray	Gel	>5cc	Monocentric, single plan, SRS only
Weyh et al. (2013) [215]	n.s.	n.s.	Gamma 3% absolute dose and 3mm DTA<br>92.9–100.0%	CBCT<br>TomoTherapy	Ion-Chamber Array	n.s.	Monocentric, small cases, no motion compensation

Supplementary table 1: Guidelines and publications for mechanical and dosimetric accuracy for stereotactic radiotherapy. References in parentheses are part of the respective report in the row above. n.s.: not specified.
